# Shenkang Injection and Its Three Anthraquinones Ameliorates Renal Fibrosis by Simultaneous Targeting IƙB/NF-ƙB and Keap1/Nrf2 Signaling Pathways

**DOI:** 10.3389/fphar.2021.800522

**Published:** 2021-12-22

**Authors:** Liang-Pu Luo, Ping Suo, Li-Li Ren, Hong-Jiao Liu, Yamei Zhang, Ying-Yong Zhao

**Affiliations:** ^1^Faculty of Life Science and Medicine, Northwest University, Xi’an, China; ^2^ School of Traditional Chinese Medicine, Southern Medical University, Guangzhou, China; ^3^Clinical Genetics Laboratory, Affiliated Hospital and Clinical Medical College of Chengdu University, Chengdu, China

**Keywords:** chronic kidney disease, shenkang injection, chrysophanol (PubChem CID: 10208), emodin, rhein (PubChem CID: 10168), oxidative stress and inflammation, lkB/NF-kB signaling pathway, Keap1/Nrf2 signaling pathway

## Abstract

Oxidative stress and inflammation are important and critical mediators in the development and progression of chronic kidney disease (CKD) and its complications. Shenkang injection (SKI) has been widely used to treat patients with CKD. Although the anti-oxidative and anti-inflammatory activity was involved in SKI against CKD, its bioactive components and underlying mechanism remain enigmatic. A rat model of adenine-induced chronic renal failure (CRF) is associated with, and largely driven by, oxidative stress and inflammation. Hence, we identified the anti-oxidative and anti-inflammatory components of SKI and further revealed their underlying mechanism in the adenine-induced CRF rats. Compared with control rats, the levels of creatinine, urea, uric acid, total cholesterol, triglyceride, and low-density lipoprotein cholesterol in serum were significantly increased in the adenine-induced CRF rats. However, treatment with SKI and its three anthraquinones including chrysophanol, emodin, and rhein could reverse these aberrant changes. They could significantly inhibit pro-fibrotic protein expressions including collagen I, α-SMA, fibronectin, and vimentin in the kidney tissues of the adenine-induced CRF rats. Of note, SKI and rhein showed the stronger inhibitory effect on these pro-fibrotic protein expressions than chrysophanol and emodin. Furthermore, they could improve dysregulation of IƙB/NF-ƙB and Keap1/Nrf2 signaling pathways. Chrysophanol and emodin showed the stronger inhibitory effect on the NF-κB p65 protein expression than SKI and rhein. Rhein showed the strongest inhibitory effect on p65 downstream target gene products including NAD(P)H oxidase subunits (p47^phox^, p67^phox^, and gp91^phox^) and COX-2, MCP-1, iNOS, and 12-LO in the kidney tissues. However, SKI and rhein showed the stronger inhibitory effect on the significantly downregulated anti-inflammatory and anti-oxidative protein expression nuclear Nrf2 and its target gene products including HO-1, catalase, GCLC, and NQO1 in the Keap1/Nrf2 signaling pathway than chrysophanol and emodin. This study first demonstrated that SKI and its major components protected against renal fibrosis by inhibiting oxidative stress and inflammation via simultaneous targeting IƙB/NF-ƙB and Keap1/Nrf2 signaling pathways, which illuminated the potential molecular mechanism of anti-oxidative and anti-inflammatory effects of SKI.

## Introduction

Organ fibrosis is a pathological extension of the normal wound healing process characterized by oxidative stress and inflammation; myofibroblast activation and migration; and excessive synthesis, deposition, and remodeling of extracellular matrix (ECM) components, mainly including collagen, fibronectin, and α-smooth muscle actin (α-SMA) (Miao et al., 2021a). A variety of pathophysiological principles is shared by many fibrotic-associated diseases, such as cirrhosis, kidney fibrosis, myocardial fibrosis, and idiopathic pulmonary fibrosis (Miao et al., 2021a). Fibrotic diseases are estimated to account for up to 50% of deaths in the developed world (Mantovani and Zusi, 2020).

Renal fibrosis, characterized by tubulointerstitial fibrosis and glomerulosclerosis, is a chronic and progressive process influencing renal functions during aging and in chronic kidney disease (CKD), regardless of the cause (Bhargava et al., 2021; Li et al., 2021; Medina Rangel et al., 2021). CKD and renal fibrosis influence approximately 26–30 million adults, and 47% of 30-year-olds will develop CKD during their lifetime in America (Humphreys, 2018). About 11% of patients with stage 3 CKD will inevitably progress to end-stage renal disease (ESRD), requiring renal replacement therapies such as dialysis and transplantation (Chauveau, 2018; Jain et al., 2019; Carta et al., 2020; Sawhney and Gill, 2020). Additionally, CKD is also one of the strongest risk factors for cardiovascular disease (Yanai et al., 2021). The costs to care for patients with CKD are two times compared with as large as ESRD costs.

In the last two decades, angiotensin-converting enzyme inhibitors (ACEIs) or angiotensin receptor blockers (ARBs) have been widely recommended clinically as a standard therapy in patients with hypertension, cardiovascular disease, and CKD (Chen et al., 2019a). These drugs could effectively reduce proteinuria levels and slow down CKD progression and prevent its complications. However, chronic administration of ACEI or ARB led to the elevated levels of angiotensin II and aldosterone, which is known as angiotensin II and aldosterone escape (Wang et al., 2018). Despite these therapies, outcomes in patients with CKD remain poor.

Natural products have been widely used for prevention and treatment of renal fibrosis (Chen et al., 2018a; Chen et al., 2018b; Yang and Wu, 2021). Shenkang injection (SKI), approved by the State Food and Drug Administration of China (CFDA) in 1999, was used to treat CKD. SKI is composed of *Rhei Radix et Rhizoma* (Dahuang), *Salviae Miltiorrhizae Radix et Rhizoma* (Danshen), *Astragali Radix* (Huangqi), and *Carthami Flos* (Honghua) (Zou et al., 2020). Dahuang possessed anti-inflammatory, anti-bacterial, anti-cancer, and anti-fibrotic effects (Wang et al., 2012). Danshen exhibited anti-inflammatory, anti-oxidative, anti-tumor, cardioprotective, neuroprotective, and anti-fibrotic effects (Wang et al., 2021a). Huangqi showed anti-inflammatory, anti-oxidative, anti-infective, anti-diabesity, anti-tumor, anti-aging, and immune-enhancing properties (Salehi et al., 2021). The extracts and isolated compounds from Honghua presented various pharmacological properties, such as anti-inflammatory, anti-thrombotic, anti-tumor, anti-diabetic, and anti-myocardial ischemic effects (Tu et al., 2015). These published literatures indicated that anti-inflammatory and anti-oxidative effects were their common pharmacological activity. Therefore, it could be speculated that their anti-inflammatory and anti-oxidative effects were associated with CKD treatment of SKI. Recently, clinical studies have demonstrated that SKI could improve renal function in CKD, peritoneal dialysis patients with chronic renal failure (CRF), and diabetic nephropathy (Zhang et al., 2017; Song et al., 2019; Wang et al., 2020a; Qin et al., 2020; Zou et al., 2020; Ma, 2021). A seminal publication has highlighted that SKI treatment protected against CRF and symptoms related to CKD following treatment with traditional Chinese medicine was 73.05 and 98.00%, respectively, in a clinical trial of 2200 patients (Qin et al., 2021). The experimental studies revealed that SKI could improve renal function and inhibit tubulointerstitial fibrosis by anti-oxidative, anti-inflammatory, and anti-apoptotic effects in unilateral ureteral obstruction mice and rats, streptozotocin-induced mice, and renal ischemia–reperfusion injury (IRI) rats (Liu, 2018; Liu et al., 2019; Zhang et al., 2020; Qin et al., 2021) as well as renal tubular cells or mesangial cells treated by transforming growth factor-β1 (TGF-β1) or high glucose (Wu et al., 2015; Xu et al., 2016; Fu et al., 2019). Mechanistically, several preliminary studies have revealed that SKI alleviated CKD and renal fibrosis by inhibiting pro-inflammatory cytokines such as interleukin-6, interleukin-1β, and tumor necrosis factor-α (TNF-α) expression (Zhang et al., 2020) and modulating TGF-β1/Smad3 and JAK2/STAT3 signaling pathways (Wu et al., 2015; Qin et al., 2021). Although SKI has been demonstrated to have anti-oxidative and anti-inflammatory effects in the treatment of CKD, little is known about its underlying oxidative stress and inflammation-associated mechanisms.

Oxidative stress and inflammation played a central role in the pathogenesis and progression of CKD (Chen et al., 2018c). Oxidative stress and inflammation are inseparably linked as they form a vicious cycle in which oxidative stress provokes inflammation by several mechanisms including activation of the nuclear factor kappa B (NF-ƙB) which leads to the activation and recruitment of immune cells, meanwhile, activation of the nuclear factor-erythroid-2–related factor 2 (Nrf2) which regulates the basal activity and coordinated induction of numerous genes that encode various anti-oxidant and phase 2 detoxifying enzymes and related proteins. In this research, a CRF rat model was induced by adenine orally, which was then administered with SKI and its bioactive components including chrysophanol, emodin, and rhein orally to determine whether they could improve CKD and slow down renal fibrosis by regulating the inhibitor of kappa B (IƙB)/NF-ƙB and Keap1/Nrf2 signaling pathways. Furthermore, we used the TGF-β1–induced human proximal epithelial cells to explore the therapeutic mechanism of SKI and its bioactive components on renal injury.

## Materials and Methods

### Chemicals and Reagents

SKI was purchased from Shijishenkang Pharmaceutical Company Ltd. (Xi’an, China). The primary antibodies including collagen I (ab34710, Abcam, United States), α-SMA (ab7817, Abcam, United States), fibronectin (ab2413, Abcam, United States), vimentin (ab92547, Abcam, United States), p-NF-ƙB p65 (13346, Cell Signaling Technology, United States), phosphorylated IƙBα (p-IκBα, 2859, Cell Signaling Technology, United States), gene cyclooxygenase 2 (COX-2, ab62331, Abcam, United States), monocyte chemotactic protein-1 (MCP-1, ab7202, Abcam, United States), inducible nitric oxide synthase (iNOS, ab178945, Abcam, United States), 12-lipoxygenase (12-LO, ab167372, Abcam, United States), p47^phox^ (ab795, Abcam, United States), p67^phox^ (ab109366, Abcam, United States), and gp91^phox^ (ab80508, Abcam, United States), Keap1 (ab196346, Abcam, United States), Nrf2 (ab31163, Abcam, United States), heme oxygenase 1 (HO-1, ab68477, Abcam, United States), catalase (ab16731, Abcam, United States), glutamate–cysteine ligase catalytic subunit (GCLC, ab190685, Abcam, United States), and NAD(P)H dehydrogenase quinone 1 (NQO1, ab28947, Abcam, United States) were purchased from Abcam Company (Cambridge, MA, United States) and Cell Signaling Technology (Danvers, MA, United States). Glyceraldehyde-3-phosphate dehydrogenase (GAPDH, 10494-1-AP) and histone H3 (17168-1-AP) were purchased from Proteintech Company (Wuhan, China).

### Extraction and Isolation of Chrysophanol, Emodin, and Rhein

SKI (10 L) was concentrated using a rotatory evaporator in vacuum to yield 2.1 kg of dry brown extract. The concentrated extract was extracted with petroleum ether (3 × 7.5 L), ethyl acetate (3 × 7.5 L), and n-butanol (3 × 7.5 L), successively. The ethyl acetate extract was chromatographed on a MCI column. Elution was performed using a solvent mixture of MeOH/H_2_O with an escalating amount of MeOH and similar fractions, identified by thin-layer chromatography, which were combined to yield five major fractions. The compounds were further isolated by the Sephadex LH-20 column, reversed-phase C-18 silica column, and semi-preparative high-performance liquid chromatography method. Finally, the compounds including chrysophanol, emodin, and rhein were identified by nuclear magnetic resonance spectrometry and reference substances.

### CRF Model and Drug Administration

Male Sprague–Dawley rats (6–8 weeks old and weighing 180–210 g) were purchased from the Central Animal Breeding House of Xi’an Jiaotong University (Xi’an, Shaanxi, China). An adenine-induced CRF model was reproduced as described in detail previously (Wang et al., 2021b; Wang et al., 2021c). In brief, the rats were divided into six groups (*n* = 8/group) including control, adenine-induced CRF, SKI-treated group with CRF (CRF + SKI), chrysophanol-treated group with CRF (CRF + CHR), emodin-treated group with CRF (CRF + EMO), and rhein-treated group with CRF (CRF + RHE). Except for the control group, other groups with CRF were orally administered adenine (200 mg/kg/d) for 3 weeks. Treatment groups were administered SKI (20 ml/kg/d), chrysophanol (30 mg/kg/d), emodin (100 mg/kg/d), and rhein (150 mg/kg/d) for 3 weeks. The body weight of each rat was measured daily. After 3 weeks, individual rats were placed in metabolic cages (1 per cage) to obtain 24-h urine collections. The rats were anesthetized with 10% urethane and then blood samples and kidney tissues were collected for clinical biochemistry and histopathological analysis. All animal care and experimental procedures were approved by the Ethics Committee for Animal Experiments of Northwest University.

### Renal Function Evaluation

The levels of creatinine, urea, uric acid, total cholesterol, triglyceride, and low-density lipoprotein cholesterol (LDL-C) in serum as well as creatinine in urine were determined using an Olympus AU6402 automatic analyzer.

### Light Microscopic Study

Light microscopy was conducted using 10% formalin-fixed, paraffin-embedded biopsies stained with hematoxylin-eosin (H&E) and Masson’s Trichrome stains, as previously described (Miao et al., 2020).

### Immunohistochemistry

The specific protein expressions were examined on paraffin sections of kidney tissues as previously described (Miao et al., 2020).

### Western Blot Analysis

All solutions, tubes, and centrifuges were maintained at 0–4°C. Cytoplasmic and nuclear proteins from kidney tissues were extracted based on our previous publication (Choi et al., 2010). Protein levels were detected using Western blotting as previously described (Miao et al., 2020). The blots were obtained using the enhanced chemiluminescence reagent, and the protein levels were normalized to the level of GAPDH or histone H3. Specific bands were analyzed using ImageJ 1.48v software.

### Statistical Analysis

The data are presented as mean ± SEM. Statistical analyses were performed using GraphPad Prism software v6.0. A two-tailed unpaired Student’s *t*-test was used for comparisons between two groups. Statistically significant differences amongst more than two groups were analyzed by one-way analysis of variance followed by Dunnett’s *post hoc* tests. *p* < 0.05 was considered significant differences.

## Results

### SKI and Its Main Components Improved the Impaired Renal Function and Injury

**FIGURE 1:**
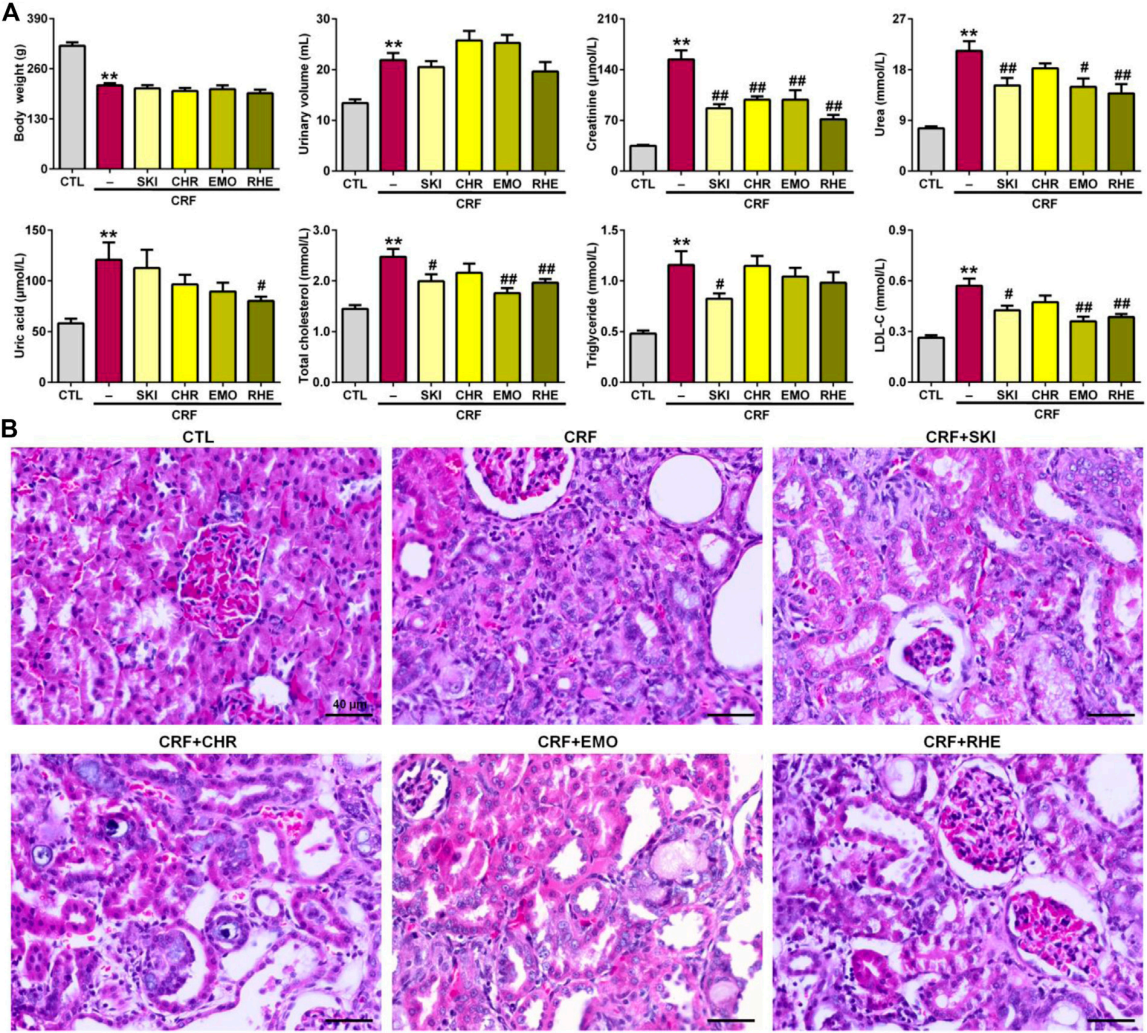
SKI and three anthraquinones including chrysophanol, emodin, and rhein improved renal function and injury in the adenine-induced CRF rats. **(A)** Body weight and urinary volume as well as clinical serum biochemistry including creatinine, urea, uric acid, total cholesterol, triglyceride, and LDL-C in the different groups. **p < 0.01 compared with the CTL group; ^#^ *p* < 0.05, ^##^ *p* < 0.01 compared with the CRF group. **(B)** Images of H&E staining of the kidney tissues in the different groups. Scale bar, 40 μm.

The final metabolite of adenine is uric acid. After adenine given by the oral gavage, excessive adenine can be oxidized to 2,8-dihydroxyadenine via an 8-hydroxyadenine intermediate by xanthine dehydrogenase. Low solubility of 2,8-dihydroxyadenine can form precipitation in the renal tubules, which led to renal injury and fibrosis. As shown in Figure 1A, intragastric adenine led to significantly decreased body weight and increased urinary volume in CRF rats, while treatment with SKI and three anthraquinones including chrysophanol, emodin, and rhein did not produce the significant changes for body weight and urinary volume. The levels of creatinine, urea, uric acid, total cholesterol, triglyceride, and LDL-C in serum were significantly increased in the adenine-induced CRF group compared with the control group. Except for uric acid, all these increases were improved by treatment with SKI. Similarly, except for triglyceride, all these increases were improved by treatment with rhein. Treatment with emodin significantly lowered the levels of creatinine, urea, TC, and LDL-C in the adenine-induced CRF group, while the levels of uric acid and triglyceride were decreased in the adenine-induced CRF group treated by emodin, but did not arrive at statistical significance. Treatment with chrysophanol only significantly lowered the creatinine levels in the adenine-induced CRF group. Compared with the control rats, H&E staining showed that the kidney tissues of the adenine-induced CRF rats showed severe inflammatory cell infiltration, tubular dilation, and interstitial fibrosis (Figure 1B). These injuries were improved by treatment with SKI and its main components including chrysophanol, emodin, and rhein. Collectively, these results demonstrated that SKI could improve the impaired renal function and ameliorate renal injury in the late stages of CKD. This effect was followed by rhein treatment. Similar results were observed in the adenine-induced CRF group treated by rhein. Furthermore, chrysophanol showed a certain renoprotective effect on adenine-induced renal function decline and damage.

### SKI and Its Main Components Ameliorated Renal Fibrosis

**FIGURE 2:**
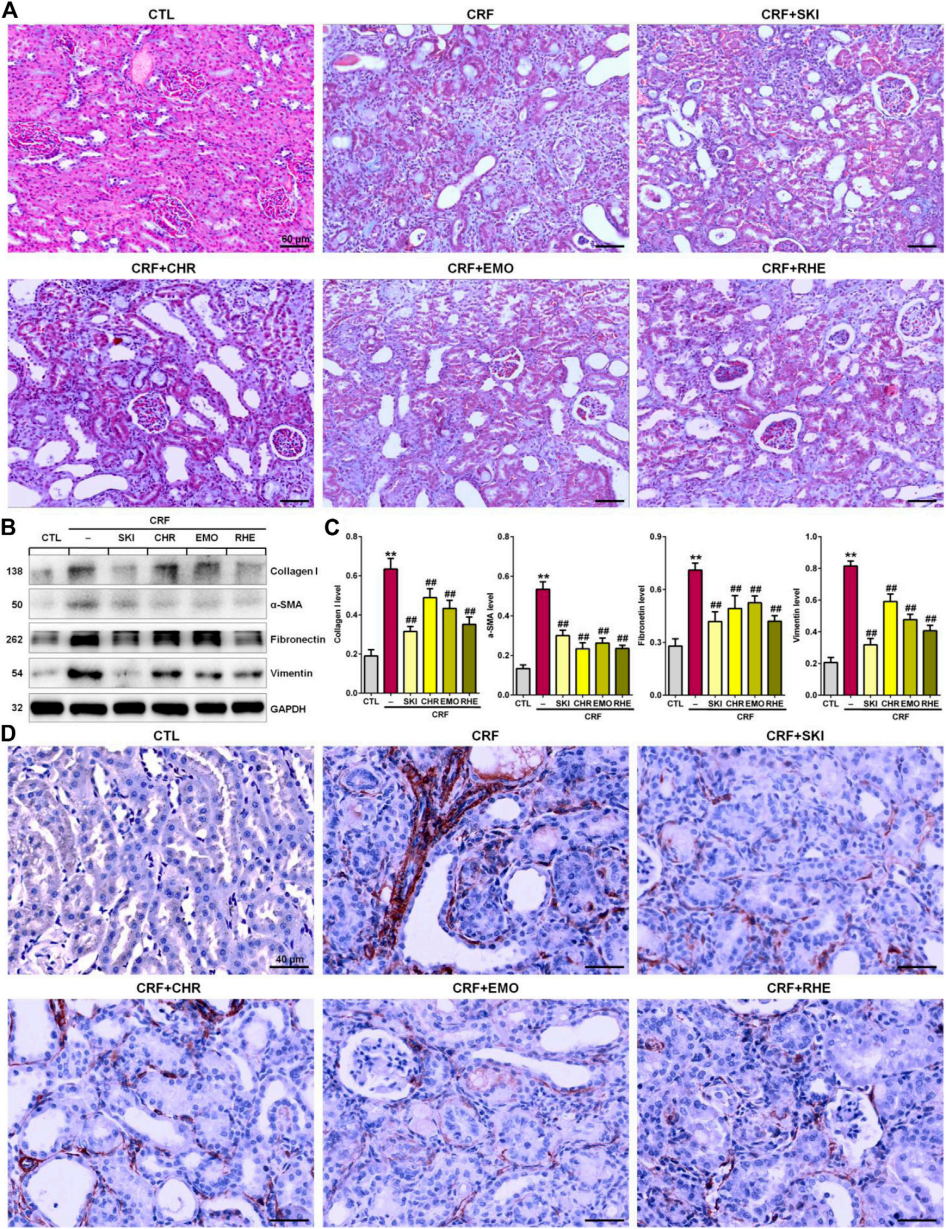
SKI and three anthraquinones including chrysophanol, emodin, and rhein ameliorated renal fibrosis in the adenine-induced CRF rats. **(A)** Images of Masson’s Trichrome staining of the kidney tissues in the different groups. Scale bar, 60 μm. **(B)** Expressions of profibrotic proteins including collagen I, α-SMA, fibronectin, and vimentin of the kidney tissues in the different groups. **(C)** Quantitative analysis of profibrotic protein expressions of the kidney tissues in the different groups. **p < 0.01 compared with the CTL group; ^##^ *p* < 0.01 compared with the CRF group. **(D)** Immunohistochemical analysis with anti–α-SMA of the kidney tissues in the different groups. Scale bar, 40 μm.

Renal fibrosis is characterized by an excessive accumulation and deposition of ECM components. As shown in Figure 2A, Masson’s Trichrome staining showed severe tubulointerstitial fibrosis in the kidney tissues of the adenine-induced CRF rats compared with the normal control rats. However, the fibrosis was improved by treatment with SKI and three anthraquinones including chrysophanol, emodin, and rhein. ECM components mainly included collagen I, collagen III, α-SMA, fibronectin, and vimentin. Therefore, we further determined the expression of pro-fibrotic proteins including collagen I, α-SMA, fibronectin, and vimentin. As shown in Figures 2B,C, the kidney tissues of the adenine-induced CRF rats showed significant upregulation of protein expression of collagen I, α-SMA, fibronectin, and vimentin compared with the control rats. However, treatment with SKI and three anthraquinones showed significant inhibitory effect on these pro-fibrotic protein expressions in the kidney tissues of the adenine-induced CRF rats. Of note, SKI and rhein showed the stronger inhibitory effect on the pro-fibrotic protein expression than chrysophanol and emodin, which was consistent with the results of clinical biochemistry and histological analyses including H&E and Masson’s Trichrome stainings. Additionally, immunohistochemistry analysis further demonstrated treatment with SKI and three anthraquinones could significantly inhibit the α-SMA expression in the kidney tissues of the adenine-induced CRF rats compared with those found in the CRF rats (Figure 2D). Of note, SKI and rhein showed the stronger inhibitory effect on the pro-fibrotic protein expression than chrysophanol and emodin. These results demonstrated that SKI and three anthraquinones protected against renal fibrosis in the adenine-induced CRF rats. Therefore, we concluded that anthraquinones might be one of the main renoprotective components of SKI.

### SKI and Its Main Components Retarded Inflammation Response

**FIGURE 3:**
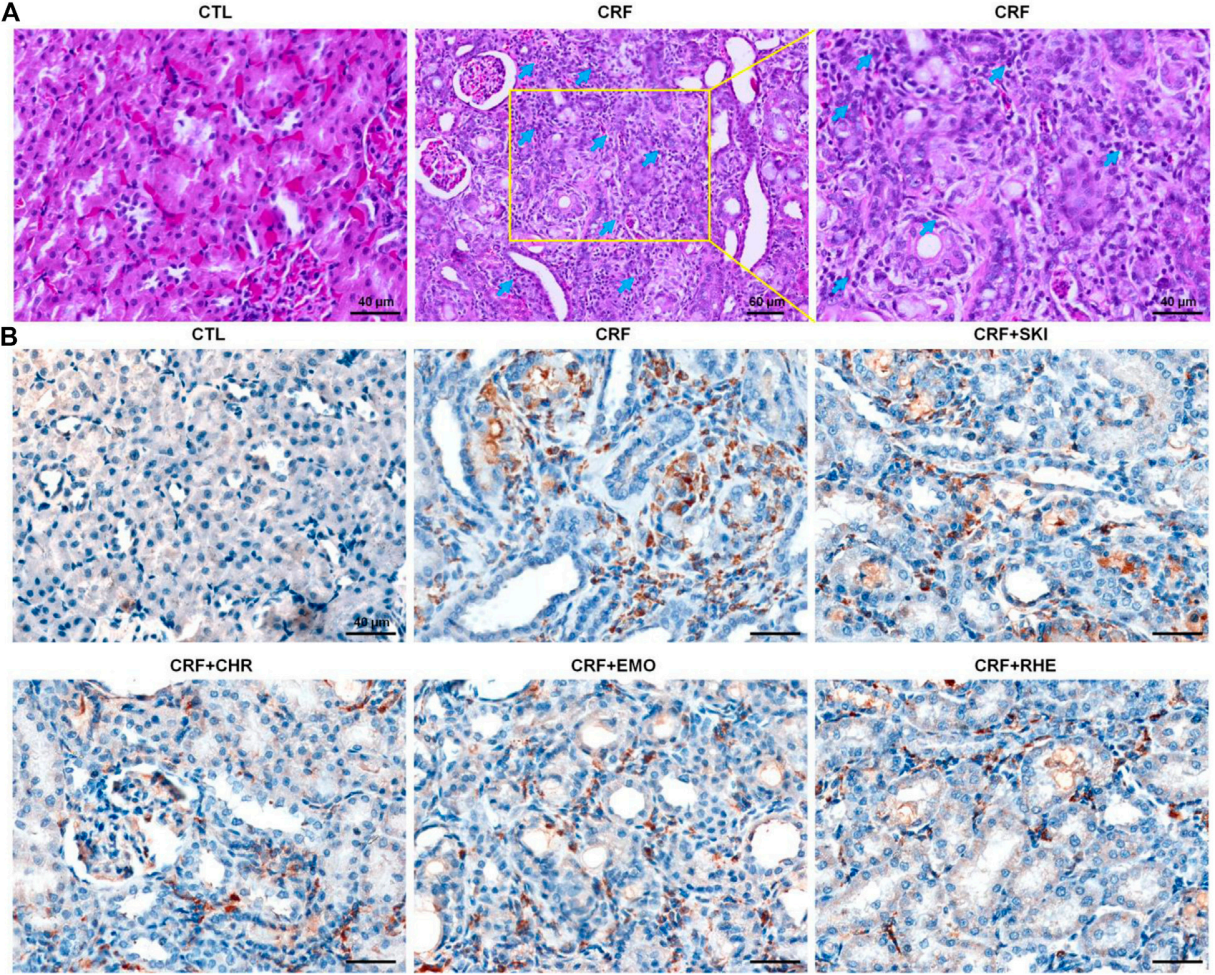
SKI and three anthraquinones including chrysophanol, emodin, and rhein inhibited inflammation in the adenine-induced CRF rats. **(A)** Images of H&E staining of the kidney tissues in the different groups Scale bar, 40 and 60 μm. Numerous inflammatory cells were indicated by arrows. **(B)** Immunohistochemical analysis with anti-CD68 of the kidney tissues in the different groups. Scale bar, 40 μm.

Histopathological examination showed that severe inflammatory cell infiltration in the renal interstitium is one of the typical characteristics in rats induced by adenine (Figure 3A). CD68 is often used as a histochemical marker of inflammation response, which was involved in the monocytes/macrophages. Therefore, we determined the anti-CD68 expression in the kidney tissues of the adenine-induced CRF rats. As shown in Figure 3B, the renal interstitium of CRF rats showed significantly increased CD68 expression compared with that of the control rats. However, treatment with SKI and three anthraquinones showed significantly decreased CD68 expression in the renal interstitium of the adenine-induced CRF rats. Collectively, these results indicated administered adenine triggered oxidative stress and inflammation. Therefore, we speculated that the molecular mechanisms of SKI and three anthraquinones against tubulointerstitial fibrosis might be associated with the activation of oxidative stress and inflammation.

### SKI and Its Main Components Ameliorated Renal Fibrosis by Inhibiting the IκB/NF-κB Signaling Pathway

**FIGURE 4:**
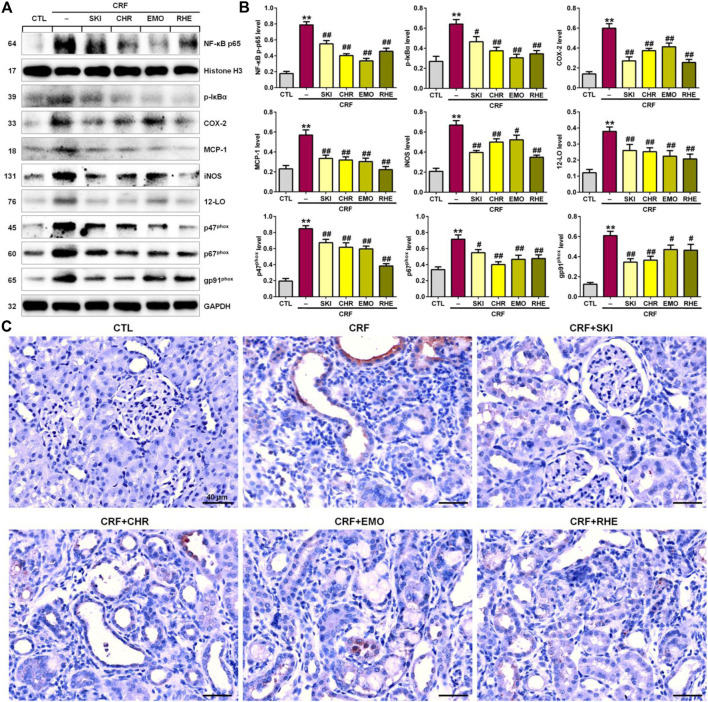
SKI and three anthraquinones including chrysophanol, emodin, and rhein inhibited the pro-inflammatory IκB/NF-κB signaling pathway in the adenine-induced CRF rats. **(A)** Protein expressions of nuclear translocation of p65 and its downstream gene products including COX-2, MCP-1, iNOS, 12-LO, and NAD(P)H oxidase subunits (p47^phox^, p67^phox^, and gp91^phox^) of the kidney tissues in the different groups. **(B)** Quantitative analysis of pro-inflammatory and pro-oxidative protein expressions of the kidney tissues in the different groups. ^**^ *p* < 0.01 compared with the CTL group; ^#^ *p* < 0.05, ^##^ *p* < 0.01 compared with the CRF group. **(C)** Immunohistochemical analysis with anti–COX-2 of the kidney tissues in the different groups. Scale bar, 40 μm.

The interplay between oxidative stress and inflammation form a vicious cycle in which oxidative stress triggers inflammation by various mechanisms such as the activation of the IƙB/NF-ƙB signaling pathway. As shown in Figures 4A,B, the kidney tissues of adenine-induced CRF rats showed significantly upregulated p-IƙB and nuclear p65 levels compared with those of the control group, which indicated the activation of the IƙB/NF-ƙB signaling pathway. This was accompanied by the significantly upregulated protein expressions of COX-2, MCP-1, iNOS, 12-LO, and NAD(P)H oxidase subunits (p47^phox^, p67^phox^, and gp91^phox^) in the kidney tissues of the adenine-induced CRF rats compared with those of the control rats. However, these upregulated expressions were inhibited in the adenine-induced CRF rats treated by SKI and three anthraquinones. Additionally, immunohistochemistry analysis demonstrated that treatment with SKI and three anthraquinones could significantly inhibit the COX-2 expression in the kidney tissues of the adenine-induced CRF rats compared with that of the CRF rats (Figure 4C). Of note, chrysophanol and emodin showed the stronger inhibitory effect on the NF-κB p65 protein expression than SKI and rhein. Rhein showed the strongest inhibitory effect on p65 downstream target gene products. Taken together, these results indicated that the inhibition of the pro-inflammatory IκB/NF-κB signaling pathway was involved in SKI and three anthraquinones against renal fibrosis.

### SKI and Its Main Components Ameliorated Renal Fibrosis by Activating the Keap1/Nrf2 Signaling Pathway

**FIGURE 5:**
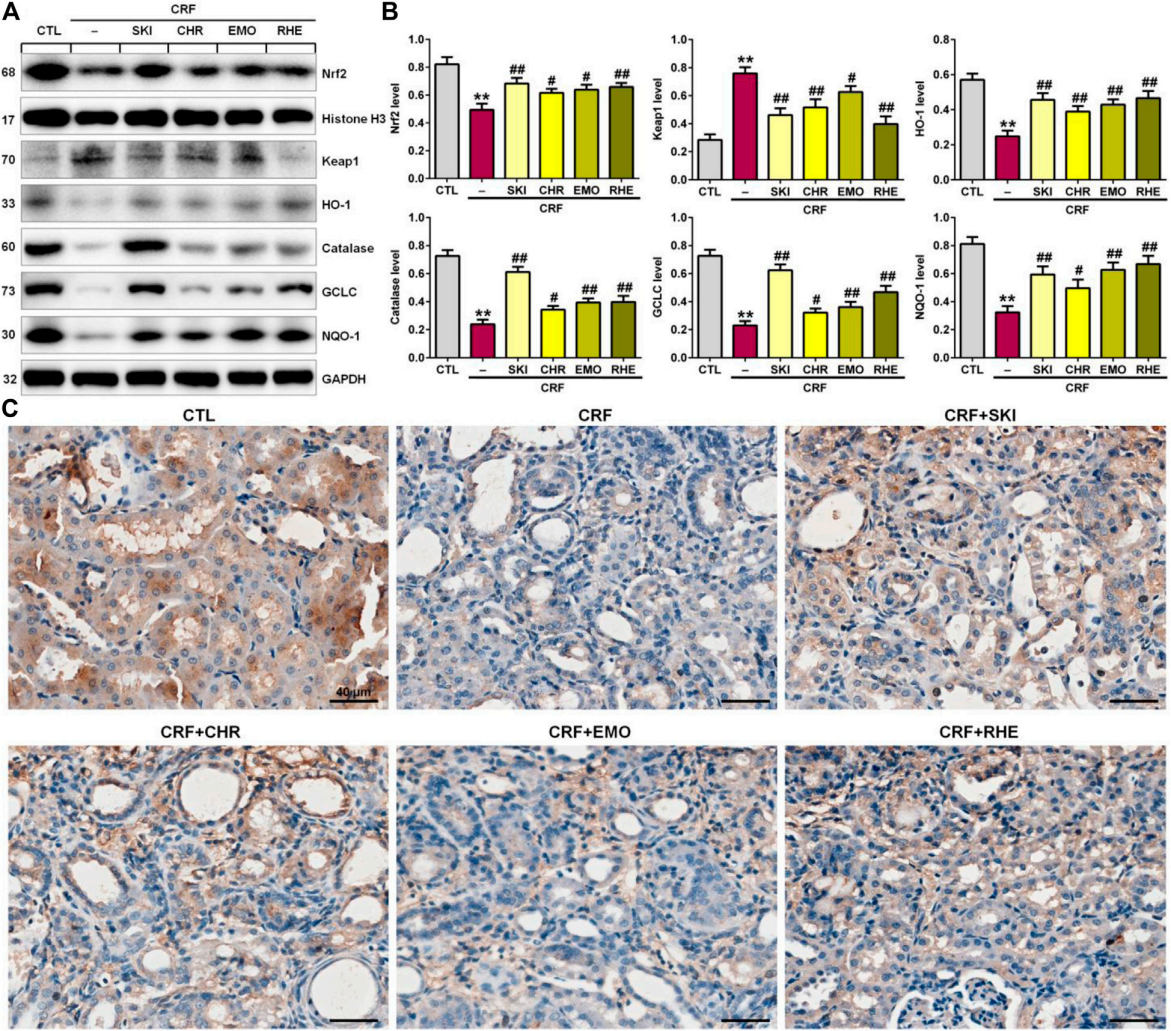
SKI and three anthraquinones including chrysophanol, emodin, and rhein activated the anti-inflammatory and anti-oxidative Keap1/Nrf2 signaling pathway in the adenine-induced CRF rats. **(A)** Protein expressions of nuclear translocation of Nrf2 and its repressor, Keap1, and its downstream gene products including HO-1, catalase, GCLC, and NQO-1 of the kidney tissues in the different groups. **(B)** Quantitative analysis of anti-inflammatory and anti-oxidative protein expressions of the kidney tissues in the different groups. ^**^ *p* < 0.01 compared with the CTL group; ^#^ *p* < 0.05, ^##^ *p* < 0.01 compared with the CRF group. **(C)** Immunohistochemical analysis with anti–HO-1 of the kidney tissues in the different groups. Scale bar, 40 μm.

Increased oxidative stress activated the expression of the endogenous anti-oxidant proteins to reduce tissue damage, which was mediated by the activation of the Keap1/Nrf2 signaling pathway. As shown in Figures 5A,B, the adenine-induced CRF rats exhibited the significantly downregulated Nrf2 protein expression and upregulated Keap1 protein expression in the kidney tissues compared with the control rats. This was accompanied by significantly downregulated Nrf2 downstream target gene products including HO-1, catalase, GCLC, and NQO-1 in the kidney tissues of rats with adenine-induced CRF. These findings point to the impaired activation of the Nrf2 pathway in this model. However, these aberrant changes were reversed in the adenine-induced CRF rats treated by SKI and three anthraquinones. Additionally, immunohistochemistry analysis showed treatment with SKI and three anthraquinones could significantly enhance the COX-2 expression in the kidney tissues of the adenine-induced CRF rats compared with that of the CRF rats (Figure 5C). Furthermore, SKI and rhein showed the stronger inhibitory effect on the significantly downregulated anti-inflammatory and anti-oxidative protein expression in the Keap1/Nrf2 signaling pathway than chrysophanol and emodin, which was consistent with the results of their effects on the pro-fibrotic protein expression including collagen I, α-SMA, fibronectin, and vimentin. Taken together, these results indicated that the activation of the anti-inflammatory and anti-oxidative Keap1/Nrf2 signaling pathway was involved in SKI and three anthraquinones against renal fibrosis in the adenine-induced CRF rats.

## Discussion

The progression of CKD and renal fibrosis, one of the biggest issues in nephrology, indicated that patients inevitably progress ESRD and require dialysis or kidney transplantation (Webster et al., 2017; Aydin et al., 2019; Van Sandwijk et al., 2019). Numerous studies have demonstrated that renal fibrosis was associated with the dysbiosis or dysregulation of gut microbiota, non-coding RNAs, renin–angiotensin system, aryl hydrocarbon receptor, IƙB/NF-ƙB, Keap1/Nrf2, TGF-β/Smad, and Wnt/β-catenin signaling pathways (Ma et al., 2018a; Chen et al., 2019b; Garg and Maurya, 2019; Zhao et al., 2019; Hu et al., 2020a; Miao et al., 2021b; Wang et al., 2021d; Wu et al., 2021; Zhou et al., 2021) as well as metabolite disorders including tryptophan metabolism and lipid metabolism (Zhao, 2013a; Zhao et al., 2015; Wang et al., 2019; Liu et al., 2021a). Further studies have demonstrated that activation of IƙB/NF-ƙB and Keap1/Nrf2 signaling pathways could mediate or crosstalk these signaling pathways in both patients with CKD and experimental research studies (Chen et al., 2017a; Chen et al., 2017b; Chen et al., 2019c). Of note, IƙB/NF-ƙB and Keap1/Nrf2 signaling pathways were the most important mediators in oxidative stress and inflammation that played a central role in the development and progression of CKD and its complications (Meng et al., 2014; Chen et al., 2016; Feng et al., 2019a). Oxidative stress was a status in which reactive oxygen species (ROS) generation surpassed the anti-oxidant defense system capacity. It led to the increased ROS production and damaged anti-oxidant capacity. Oxidative stress and inflammation were inseparably linked, as each begets and amplifies the other.

The activation of NF-ƙB and the impairment of Nrf2 were the most important pro-inflammatory and anti-inflammatory signals, respectively. The NF-ƙB activation mediated the expression of pro-inflammatory cytokines and chemokines, and oxidative stress evoked recruitment and activation of leukocytes and resident cells, thus triggering inflammation (Miao et al., 2021a). Although oxidative stress and inflammation had a central role in progression of CKD, ACEI and ARB have been used as first-line drugs for treatment of CKD and its complications. This led to the contradiction between the underlying pathomechanism elucidation and the treatment of CKD patients. Therefore, developing the agents to target oxidative stress and inflammation is necessary for the effective treatment of CKD patients.

The adenine diet led to severe CRF due to adenine-derived very low-soluble 2,8-dihydroxyadenine in the renal tubule, which induced tubulointerstitial nephritis, characterized by gross swelling, kidney discoloration and deformity, urinary concentrating ability loss (polyuria), azotemia (increased serum urea), anemia, hypertension, and minimal proteinuria (Zhao et al., 2014). The histopathological results showed tubulointerstitial damage including extensive inflammatory cell infiltration, tubular dilation, and fibrosis in the kidney tissues. Severe interstitial inflammatory cell infiltration was one of the most typical pathological features in the kidney tissues of adenine-induced CRF rats. Substantial evidence has demonstrated that many natural products, such as *Rhubarb*, *Astragalus*, and *Polyporus umbellatus*, showed the renoprotective activity by anti-oxidative and/or anti-inflammatory effects (Wang et al., 2012; Zhao, 2013b; Zhang et al., 2014; Shahzad et al., 2016; Liao et al., 2017). Although, the exact mechanisms for these natural products have not been revealed, it has been suggested that they may possibly possess anti-oxidant and/or anti-inflammatory activity. Our current findings demonstrated rats with CRF showed the upregulating protein expression of p-IƙBα and nuclear p65 indicating NF-ƙB activation in kidney tissues, meanwhile, this was accompanied by the upregulating protein expression of COX-2, MCP-1, iNOS, 12-LO, and NAD(P)H oxidase subunits (p47^phox^, p67^phox^, and gp91^phox^) in the kidney tissues of the adenine-induced CRF rats. However, these upregulating expressions were inhibited by treatment of SKI. These results were in agreement with previous studies of natural products, such as *Poria cocos* and *Polyporus umbellatus* as well as their components including poricoic acid A, poricoic acid ZM, poricoic acid ZP, and ergone against renal fibrosis by targeting IƙB/NF-ƙB and Keap1/Nrf2 signaling pathways (Feng et al., 2019b; Chen et al., 2019d; Chen et al., 2019e; Chen et al., 2019f; Wang et al., 2020b). Both clinical and experimental studies have demonstrated that SKI could improve renal function in CKD. Several previous publications have highlighted that SKI retarded renal fibrosis by inhibiting levels of interleukin-6, interleukin-1β, and TNF-α (Zhang et al., 2020) and modulating TGF-β1/Smad3 and JAK2/STAT3 signaling pathways (Wu et al., 2015; Qin et al., 2021). Another study has been demonstrated that treatment with SKI could inhibit the protein expression of NF-ƙB at both mRNA and protein levels in kidney tissues of renal ischemia–reperfusion injury mice with DN induced by high-fat diet and streptozocin (Liu, 2018). Little was known about its underlying anti-oxidative and anti-inflammatory mechanism. Our findings suggested that SKI retarded renal fibrosis by inhibiting the activation of the IƙB/NF-ƙB signaling pathway. Therefore, our current works and that of others suggested that effective inhibition of activated oxidative stress and inflammation via the IƙB/NF-ƙB signaling pathway retarded CRF progression.

In the bioactive fraction of ethyl acetate extract of SKI, we identified three anthraquinones including chrysophanol, emodin, and rhein that were major and bioactive components of *Rheum officinale*, which has been demonstrated to improve CKD and renal fibrosis (Wang et al., 2012; Zhang et al., 2018; Zeng et al., 2021). Compared with chrysophanol and emodin, rhein showed a strong inhibitory effect on renal fibrosis. Although rhein has been widely demonstrated to protect against renal fibrosis (Zeng et al., 2014; Hu et al., 2019; He et al., 2020; Wu et al., 2020; Yu et al., 2020), only two previous studies have reported that rhein protected against renal fibrosis by inhibiting the NF-ƙB p65 protein expression (Liu et al., 2021b) and lincRNA-COX2/miR-150-5p/STAT1 axis (Hu et al., 2020b). Similarly, a number of publications have demonstrated that emodin retarded renal fibrosis by modulating several pathways, such as TGF-β/Smad, TGF-β/BMP-7, PI3K/Akt/GSK-3β, and Bax/caspase-3 signaling pathways (Jing et al., 2017; Ma et al., 2018b; Yang et al., 2020; Liu et al., 2021c). Furthermore, several studies have demonstrated the inhibitory effect of emodin on renal fibrosis by suppressing the NF-ƙB p65 protein expression (Lu et al., 2020) or the levels of ROS, TNF-α, and interleukin-6 (Chen et al., 2017c; Jing et al., 2017). So far, no publication demonstrated the renoprotective effect of chrysophanol through modulating the IƙB/NF-ƙB signaling pathway. Recently, two publications have demonstrated that chrysophanol protected against renal fibrosis by the TGF-β/Smad signaling pathway (Dou et al., 2020; Guo et al., 2020). Therefore, our study demonstrated that the inhibition of the activated IƙB/NF-ƙB signaling pathway might be the underlying molecular mechanism of anti-oxidant and anti-inflammatory bioactivities of both SKI and three anthraquinones against renal fibrosis.

Compared with pro-inflammatory system, the natural anti-oxidant defense system contains many ROS scavenger molecules from exogenous dietary and endogenous components, anti-oxidant enzymes and substrates, and phase 2 detoxifying enzymes (Cuadrado et al., 2019). Each component contributes to their specific function and works in a collaborated way with the other components to exert their protective effects against tissue damage and dysfunction. Under physiological milieu, oxidative stress elicited increasing endogenous anti-oxidant and cytoprotective proteins and enzymes to restrain dysfunction and tissue damage (Cuadrado et al., 2019). This process was induced by the activation of the Nrf2 which plays a central role in the basal activity and coordinated regulation of about 250 genes such as HO-1, GCLC, NQO1, catalase, superoxide dismutase, thioredoxin, and glutamate–cysteine ligase (Chen et al., 2017a; Cuadrado et al., 2019). Our findings first demonstrated SKI treatment could upregulate nuclear Nrf2 protein expression and downregulate Keap1 protein expression in the kidney tissue of CRF rats, which was accompanied by upregulating Nrf2 downstream target gene products. To date, no publication demonstrated the renoprotective effect of SKI through activating the Keap1/Nrf2 signaling pathway. Our current findings first point to the beneficial effects of SKI on the impaired activation of the Keap1/Nrf2 pathway in the adenine-induced CRF rats.

Although increasing evidence has reported that chrysophanol, emodin, and rhein could improve many refractory diseases by the activation of the impaired Keap1/Nrf2 signaling pathway, and few studies demonstrated the renoprotective effect of chrysophanol, emodin, and rhein by regulating the Keap1/Nrf2 signaling pathway. Two previous *in vitro* studies have demonstrated that emodin could increase the activities of anti-oxidant enzymes such as catalase, glutathione peroxidase, superoxide dismutase, glutathione reductase, and glutathione S-transferase in the hypoxia/reoxygenation-induced HK-2 cells or cisplatin-induced human kidney HEK 293 cells (Waly et al., 2013; Chen et al., 2017c). Another *in vivo* experiment has demonstrated that emodin significantly inhibited the decreased renal cortical glutathione levels and superoxide dismutase activity in the cisplatin-induced nephrotoxicity rats (Ali et al., 2013). However, no publication demonstrated the renoprotective effect of chrysophanol and rhein through modulating the Keap1/Nrf2 signaling pathway. Therefore, our study revealed that activation of the impaired Keap1/Nrf2 signaling pathway might be also a potential molecular mechanism of anti-oxidant and anti-inflammatory bioactivities of both SKI and three anthraquinones against renal fibrosis.

Collectively, our current study first elucidated that SKI and its main components including chrysophanol, emodin, and rhein protected against renal fibrosis by inhibiting oxidative stress and inflammation via simultaneous targeting pro-inflammatory IƙB/NF-ƙB and anti-inflammatory Keap1/Nrf2 signaling pathways, which revealed the underlying molecular mechanism of SKI and its main components against renal fibrosis. These findings uncovered the potential effective material basis and molecular mechanism of the renoprotective effect of SKI, which will pave the way for discovery of lead compounds against renal fibrosis by inhibiting oxidative stress and inflammation via targeting the redox pathway.

## Conclusion

This study first demonstrated that SKI and its components including chrysophanol, emodin, and rhein protected against renal fibrosis. Mechanistically, this study revealed the potential molecular mechanism of the anti-oxidative and anti-inflammatory effects of SKI by inhibiting oxidative stress and inflammation via simultaneous targeting IƙB/NF-ƙB and Keap1/Nrf2 signaling pathways.

## Data Availability

The raw data supporting the conclusion of this article will be made available by the authors, without undue reservation.
